# Effects of metabolic syndrome on bone mineral density in postmenopausal Turkish women

**DOI:** 10.4314/ahs.v23i3.13

**Published:** 2023-09

**Authors:** Zeynep T Bahtiyarca, Azize Serçe

**Affiliations:** 1 Department of Physical Medicine and Rehabilitation, University of Health Science, Diskapi Yildirim Beyazit Education and Research Hospital, Ankara, Turkey; 2 Department of Algology, Ankara Training and Research Hospital, Ankara, Turkey

**Keywords:** Postmenopausal women, bone mineral density, metabolic syndrome, osteoporosis

## Abstract

**Objective:**

In this study, we aimed to determine the potential effects of metabolic syndrome (MetS) and its components on bone mineral density (BMD) in the lumbar spine and femoral neck in postmenopausal Turkish women.

**Patients and methods:**

193 postmenopausal women were included in this study. Anthropometric measurements, biochemical blood tests, and T-scores of BMD in the lumbar spine (L1–L4) and femoral neck were recorded. The participants were divided into two groups according to the bone mineral density (BMD) results as osteoporosis group (Group 1, n=109) who had a T-score>−2.5 at the spine or/and femoral neck, and the control group (Group 2, n=84) who had T-score>−2.5 at the spine or/and femoral neck. MetS and its components were screened using the criteria of the Adult Treatment Panel III (ATP III) and National Cholesterol Education Program (NCEP). The effects of the MetS components on T-scores of BMD at the femoral neck and lumbar spine were evaluated by partial correlation test and multiple regression analysis.

**Results:**

MetS was detected in 58 (30.1%) participants. The prevalence of MetS was significantly higher in Group 2 compared to Group 1 (39.3% vs 22.9%, p=0,014). Among the MetS components, especially abdominal obesity showed a significant positive correlation with T-scores of BMD at the femoral neck and spine. A weak but significant correlation was also observed with systolic and diastolic blood pressure, serum triglyceride levels, and fasting blood glucose (FBG). Multiple regression analysis revealed an association between waist circumference and BMD at both femoral neck and spine, and also between serum triglycerides, systolic and diastolic blood pressure, and spine BMD.

**Conclusion:**

Our findings support that MetS is associated with increased BMD at the femoral neck and spine in postmenopausal women. A significant positive association was observed among the MetS components, especially with abdominal obesity, and also a weak positive association with serum triglycerides, and systolic and diastolic blood pressure.

## Introduction

Osteoporosis and metabolic syndrome (MetS) are two important public health problems in the aging population due to the increasing prevalence of these disorders worldwide, including in Turkey. The estimated prevalence of MetS in Turkish adults aged 40 years or over has reached 53% 1. This data indicates that a large number of people in Turkey suffer from MetS. This cohort study also showed that MetS is common in 45.1% of men and 54.5% of women 1. The incidence of MetS increases in the postmenopausal period mainly due to the decrease in the levels of sex hormones 2. Many studies have supported that menopause is a predisposing factor for MetS in women 3, 4. The main components of MetS are abdominal fat accumulation, dyslipidemia (poor high-density lipoprotein and/or increased triglyceride levels), high blood pressure, and hyperglycemia. It is required the presence of at least three of the five components listed above for diagnosis of MetS 5. Each component of MetS can affect bone mineral density and bone turnover by various mechanisms 6.

Bone mineral density (BMD) is a well-known measurement for determining bone mass and is also used to diagnose and determine fracture risk 7. Osteoporosis is defined as a decrease in BMD of 2.5 standard deviations (SD) or more below the mean for young healthy persons, according to the World Health Organization (WHO) criteria 8. The prevalence of osteoporosis at the femoral neck in Turkish women over the age of 50 years or more was 33.3% 9. Fragility fractures with low-energy trauma are the most common clinical result of osteoporosis. More than 40% of postmenopausal women are expected to experience a fracture at some point in their lives 10.

However, while MetS and osteoporosis were previously thought to be unrelated conditions, studies have shown that both share a variety of nutritional, endocrine, and genetic factors 6. Increasing evidence in the literature has supported the link between MetS and its components on BMD, but the results are inconsistent 11, 12. Therefore, we aimed to determine the potential effects of MetS and its components on BMD at the lumbar spine and femoral neck in postmenopausal Turkish women.

## Materials and methods

### Study design and participants

This cross-sectional study was conducted in a state hospital's Physical Medicine and Rehabilitation department between October 2020 and March 2021. A total of 193 postmenopausal women referred to the outpatient clinic for BMD testing were included in the study. Verbal and written informed consent were taken from all participants. Inclusion criteria included postmenopausal women who (i) had at least one year of menopausal experience, (ii) underwent BMD measurement by dual-energy x-ray absorptiometry (DEXA) for the assessment of osteoporosis, and (iii) accepted anthropometric measurements and blood tests required for MetS screening. Women were excluded from the study if they (i) were in the perimenopausal period, (ii) had missing BMD test data, (iii) had disorders affecting their bone metabolism, such as Paget's, hyperparathyroidism, (iv) had a renal or hepatic failure, (v) had metal implants in lumbar spine or femur, (vi) were undertaken in medications influencing bone mass such as glucocorticoids, thyroid supplements. Participants were divided into two groups according to their BMD measurements. Women with a T-score of −2.5 or less in the femoral neck or lumbar spine (L1–L4) were included in the osteoporosis group (Group1, n=109), women with a T -score above −2.5 on these sides were included in the control group (Group 2, n=84). Research protocols were followed by the Declaration of Helsinki and local ethics committee approval was obtained for the study.

### Descriptive data, anthropometry, blood samples

The demographic characteristics and personal medical histories of the participants were recorded. Risk factors for osteoporosis and physical activity status according to the Short Form of International Physical Activity Questionnaire (IPAQ–SF) were also questioned. The IPAQ-SF evaluates self-reported physical activity over the previous seven days. When physical activity level is classified, it is divided into three categories: ‘inactive’, ‘minimally active,’ and ‘highly active’. The weight and height of the participants were measured in light home wear without shoes. Body mass index (BMI) was calculated by multiplying the square of the height in meters by the weight in kilograms. Waist circumference was measured with a flexible tape measure mid-level between the iliac crest and the lowest rib when participants were standing at the end of an exhalation. Blood pressure was measured when participants were seated after resting for 10-minutes. Blood biochemical tests including fasting blood glucose (FBG), HDL cholesterol (HDL-C), triglycerides, 25-hydroxyvitamin D3, serum electrolytes, parathormone, and thyroid-stimulating hormone (TSH) were collected from all participants after a minimum of 10 hours of fasting.

### BMD measurements, fragility fracture risk

BMD was measured at the femoral neck and lumbar spine utilizing X-ray absorptiometry with dual-energy (Discovery A series, Hologic QDR). The BMD results were categorized using WHO standards. Women with a T-score of −2.5 or less in the femoral neck or lumbar spine were classified to have osteoporosis, osteopenia as −2.5 < T-score <−1, and normal as ≥−1 according to WHO criteria 8. Participants were divided into 2 groups: women with osteoporosis as a group and women with osteopenia and normal BMD as a group.

Fragility fracture risks of the participants were calculated with the Fracture Risk Assessment Tool (FRAX) which is a software that computes a ten-year risk of significant osteoporotic fractures (wrist, humerus, spine, clinical, or hip fracture) and the fracture risk of the hip for 10 years.

### Definition of MetS

Adult Treatment Panel III (ATP III) criteria of the National Cholesterol Education Program (NCEP) was used for MetS screening. Participants were diagnosed with MetS according to the NCEP-ATP III definition if three or more of the abnormalities listed below were present: abdominal obesity (waist circumference > 88cm), high blood pressure (systolic blood pressure ≥ 135mmHg and/or diastolic blood pressure ≥ 85mmHg, or if they were on anti-hypertensive medications), high FBG (≥110mg/dL, or if they were on anti-diabetic medications), dyslipidemia including hypertriglyceridemia (serum triglyceride level ≥ 150mg/dL), low HDL-C (< 50 mg/dL) 13.

### Statistical analysis

Statistical Package for Social Science (SPSS) version 20.0 software (IBM Corporation, Chicago, IL) was used to perform all statistical analyses. Categorical variables, as well as, other discrete and continuous variables were represented in percentage and number, and median (min-max), respectively, while variables with normal distribution were represented in mean±standard deviation (SD). The Kolmogorov-Smirnov test was used for data distribution analysis. To compare the descriptive and clinical features of the participants according to osteoporosis status, the chi-square test and Fisher's exact tests were used. Continuous and non-parametric variables were compared using the Mann-Whitney U test. The Pearson correlation coefficient (r) was used to determine the correlation between MetS components and T scores of BMD at the femoral neck and spine. R 0–0.25 was regarded as a weak correlation, 0:25 to 0:50 as a weak-to-moderate correlation, 0.50–0.75 as a strong correlation, and 0.75–1 was regarded as a very strong correlation. To analyse the relationship between BMD and MetS components, multiple linear regression analysis was used. A p-value of less than 0.05 was found to be statistically significant.

## Results

A total of 193 postmenopausal women participated in the study. The mean age of the participants was 67,69±8.16. Most of the participants were overweight, with a median BMI of 30.9. Osteoporosis was present in 109 women based on T-scores of BMD at the femoral neck or lumbar spine. Participants with osteoporosis were included in Group 1, and those without osteoporosis were included in Group 2. The prevalence of MetS was 30.1% in the entire study population. MetS prevalence was greater in Group 2 compared to Group 1 (39,3% vs 22,9%, p=0.014). There were also statistically significant differences in the following variables between the two groups; weight, BMI, T-scores of BMD at the lumbar spine (L1–L4) and femoral neck, the 10-year risk of major osteoporotic or hip fracture according to FRAX, pain scores. As expected, T-scores of BMD at the lumbar spine and femoral neck were significantly lower (p<0.001), while the 10-year risk of major osteoporotic fracture or hip fracture and visual analog scale scores for back pain were higher in Group 1 than Group 2 (p=0.002, <0.001, 0.006, respectively). Physical activity levels according to IPAQ-SF were similar between the two groups (p=0.486). Descriptive data and clinical features of the participants are shown in detail in Table 1.

**Table 1:** Demographic and clinical characteristics of the participants

Variables		Total (n=193)	Group-1 (n=109)	Group-2 (n=84)	P-value
Age (years)		67,69±8,16	67,47±7,85	67,95±8,53	0,898
Weight (kg)		75(43–111)	69 (43–95)	81 (55–111)	<0,001*
Height (cm)		155 (139–170)	154,5 (143–168)	155 (143–170)	0,183
Body mass index (kg/m^2^)		30,9 (18,14–49,30)	28,7 (18,14–42,20)	32 (22,40–49,30)	<0,001*
Marital status	Single	5 (2,6)	1 (20)	4 (80)	0,169
	Married	188 (97,4)	108 (57,4)	80 (42,6)	
Educational level	Unschooled	106 (54,9)	60 (56,6)	46 (43,4)	0,988
	Primary school	75 (38,9)	42 (56)	33 (44)	
	High school /University	12 (6,2)	7 (58,3)	5 (41,7)	
Occupation	Housewife	185 (95,9)	103 (55,7)	82 (44,3)	0,470
	Worker	8 (4,1)	6 (75)	2 (25)	
Type of menopause	Natural	160 (82,9)	88 (55)	72 (45)	0,362
	Surgical	33 (17,1)	21 (63,6)	12 (36,4)	
Time since menopause (years)		22 (1–49)	23 (2–49)	20 (1–43)	0,622
Number of pregnancies		5 (0–11)	5 (0–11)	4 (0–10)	0,010
Comorbidities**	Yes	135 (69,9)	75 (55,6)	60 (44,4)	0,694
	No	58 (30,1)	34 (58,6)	24 (41,4)	
Smoking	Yes	9 (4,7)	7 (77,8)	2 (22,2)	0,304
	No	184 (95,3)	102 (55,4)	82 (44,6)	
Alcohol intake (3 or more units/day)	Yes	-	-	-	-
	No	193	109	84	
History of vertebral fracture	Yes	27 (14)	19 (70,4)	8 (29,6)	0,116
	No	166 (86)	90 (54,2)	76 (45,8)	
History of peripheral fracture	Yes	48 (24,9)	25 (52,1)	23 (47,9)	0,479
	No	145 (75,1)	84 (57,9)	61 (42,1)	
Maternal hip fracture	Yes	11 (5,7)	5 (45,5)	6 (54,5)	0,537#
	No	182 (94,3)	104 (57,1)	78 (42,9)	
Bone mineral density	L1-L4 T-score	−2,43±0,99	−3,02± 0,81	−1,65±0,58	<0,001*
	Femoral neck T-score	−1,82±0,85	−2,18±0,89	−1,37±0,54	<0,001*
FRAX	10-year risk of major osteoporotic fracture	11 (3,1–42)	13 (4,2–42)	9,4 (3,1–35)	0,002*
	10-year risk of hip fracture	2 (0,8–33)	2,7 (0,8–33)	1,4 (0,1–17)	<0,001*
Visual analog scale for back pain		30 (0–80)	40 (0–70)	30 (10–80)	0,006*
Metabolic syndrome	Yes	58 (30,1)	25 (43,1)	33 (56,9)	0,014*
	No	135 (69,1)	84 (62,2)	51 (37,8)	
IPAQ-SF	İnactive	58 (30,1)	29 (50)	29 (50)	0,486
	Minimally active	112 (58)	66 (58,9)	46 (41,1)	
	Highly active	23 (11,9)	14 (60,9)	9 (39,1)	

Values are mean±SD, median (min-max) or percentage (n, %).

* P-values statistically significant (p < 0.05) are shown in bold.

** Comorbidities include hypertension, diabetes mellitus, hypothyroidism, and coronary heart disease. FRAX: Fracture risk assessment tool. IPAQ-SF: International physical activity questionnaire short form. Group-1: Osteoporosis group, Group-2: Control group.

Cardiometabolic variables including waist circumference and systolic and diastolic blood pressure were significantly lower in Group 1 than in Group 2 (p<0.001, <0.001, 0.005 respectively). Biochemical blood tests and cardiometabolic variables of the participants are shown in detail in Table 2. The correlation between MetS components and BMD at the spine and femoral neck T-scores of participants are seen in Table 3. Among the MetS components, waist circumference showed a significant positive correlation with T-scores of BMD at the femoral neck and spine. A weak but significant positive correlation was also observed with systolic and diastolic blood pressure, serum triglycerides, and FBG. Multiple regression analysis confirmed the correlation between waist circumference and BMD at the femoral neck and spine. In addition, as seen in Table 4, a positive significant relationship was demonstrated between serum triglycerides, systolic and diastolic blood pressure and spine BMD.

**Table 2:** Cardiometabolic variables and biochemical blood tests of the participants

Variables	Total (n=193)	Group-1 (n=109)	Group-2 (n=84)	P-value
Waist circumference (cm)	92,36±16,43	88,19±15,8	97,76±15,72	<0,001*
SBP (mm/hg)	124,19±11,84	123,16±10,87	129,40±12,18	<0,001*
DBP (mm/hg)	77,51±7,55	76,19±7,51	79,22±7,30	0,005*
Fasting blood glucose (mg/dl)	98 (61–435)	96 (62–435)	101 (61–247)	0,081
HDL-C (mg/dl)	49,5±9,78	49,01±8,16	50,32±11,55	0,381
Triglyceride (mg/dl)	123 (57–750)	119 (57–750)	127 (74–350)	0,361
25(OH)D3 (ng/ml)	15,4 (3–42)	15 (3–36,2)	15,7 (3–42)	0,312
Calcium (mg/dl)	9,5 (8–11,32)	9,43 (8,50–11,32)	9,5 (8–10,39)	0,456
Phosphate (mg/dl)	3,40 (2,05–5,37)	3,22 (2,05–4,72)	3,5 (2,6–5,37)	<0,001*
TSH (mlU/L)	1,5 (0,02–34,66)	1,5 (0,2–34,66)	1,57 (0,02–10,40)	0,901
PTH (pg/ml)	52,43 (18–209)	52,55 (18–209)	52,43 (22,7–130)	0,763
Uric acid (mg/dl)	4,5 (1,97–10,6)	4,2 (1,97–8,20)	4,75 (2,6–10,6)	0,901

Values are median (min-max) or mean±SD.

* P- values with statistical significance (p < 0.05) are shown in bold. Group-1: Osteoporosis group, Group-2: Control group. SBP: Systolic blood pressure, DBP: Diastolic blood pressure, HDL-C: High-density lipoprotein cholesterol, TSH: Thyroid-stimulating hormone, PTH: Parathormone, 25(OH)D3: 25-hydroxyvitamin D3.

**Table 3:** Correlation analysis between MetS components and BMD scores in participants

		Bone mineral density	

		Femoral neck T-score	Lumbar spine (L1-L4)T-score

S. No	Variables	r (p)	r (p)
		0,375 **(<0,001)**	0,340 **(<0,001)**
1	Waist circumference (cm)		

		0,205 **(0,004)**	0,253 **(<0,001)**
2	SBP (mm/hg)		

		0,150 **(0,038)**	0,098(0,176)
3	DBP (mm/hg)		

		0,100 (0,166)	0,160 **(0,027)**
4	Fasting blood glucose (mg/dl)		

		0,036 (0,616)	−0,080 (0,266)
5	HDL-C (mg/dl)		

		−0,070 (0,333)	0,164 **(0,023)**
6	Triglyceride (mg/dl)		

r: Pearson correlation coefficient. P-values with statistical significance (p < 0.05) are shown in bold. A log transformation was applied before analysis for TG and FBG. SBP = systolic blood pressure, DBP = diastolic blood pressure, HDL-C = high-density lipoprotein cholesterol

**Table 4:** Multiple regression analysis of the effects of MetS components on T-scores of BMD at the femoral neck and lumbar spine

Bone mineral density								

	Femoral neck T-score					Lumbar spine T-score		

Risk factors	B	SE	β	P	B	SE	β	P
WC	0,020	0,004	0,373	**<0,001**	0,018	0,004	0,306	**<0,001**

SBP	0,009	0,007	0,121	0,185	0,020	0,008	0,240	**0,009**

DBP	−0,005	0,010	−0,040	0,635	0,025	0,011	0,190	**0,017**

FBG	<0,001	0,001	0,031	0,660	<0,001	0,001	−0,017	0,815

HDL-C	0,010	0,006	0,111	0,127	0,007	0,007	0,071	0,332

TG	<0,001	0,001	0,015	0,837	0,002	0,001	0,174	**0,027**

B; unstandardized beta, SE; standard error, β standardized beta. A log transformation was applied before analysis for TG and FBG. P-values with statistical significance (p < 0.05) are shown in bold WC; waist circumference, SBP; systolic blood pressure, DBP; diastolic blood pressure, TG; triglyceride, HDL-C; high-density lipoprotein cholesterol, FBS; fasting blood sugar.

## Discussion

In this study, MetS were significantly associated with greater BMD and a lower prevalence of osteoporosis. Our findings support a significant positive correlation between MetS and BMD at the spine and hip in post-menopausal women. Among MetS components, this positive correlation was observed especially with abdominal obesity, and a weak but significant positive association was also observed with serum triglycerides, systolic and diastolic blood pressure.

The MetS prevalence in our study population was 30.1% which is lower than the estimated prevalence of MetS (54.5%) in Turkish women aged 40 years or over 1. Since it was a hospital-based study, the number of patients was limited. Furthermore, as the study population consisted of volunteer participants for health screening and participants referred by a physician, these individuals may be more conscious of their health and therefore the prevalence of MetS was lower than the prevalence in the general Turkish population. Its worldwide prevalence can be estimated as 21.7% (6.1–58%) 14. There are varying prevalence rates from different countries and ethnic populations. The prevalence of MetS was 27.65% in South America, 27.93% in North America, 10.47% in Europe, 21.27% in Asia, and 16.04% in Africa 14. There are fewer cohort studies among postmenopausal women. In studies from different countries, the prevalence of MetS in post-menopausal women ranges between, 41.4% in Spain 15, 22.2% in Brazil 2, 32.1% in Taiwan 16, 16.9% in Thailand 17 to 31.0% in Iran 18 and 55.5% in İndia 19. Our prevalence rate was consistent with Iran and Taiwan. Studies have also confirmed that the prevalence of MetS was higher in women in the postmenopausal period than in the premenopausal period 2, 3. The prevalence of MetS rises with age and reaches 64.4% in women aged 80–89 years 20. In most Middle Eastern countries, the prevalence of MetS was much higher among women than men 21.

Increasing evidence has supported that metabolic abnormalities affect bone health. In this study, we observed a significant positive correlation between MetS and BMD at the spine and hip in postmenopausal women. There are many studies in the literature on different populations that indicated this positive correlation consistent with our results. In a meta-analysis by Xue et al., it was reported that the BMD values at the femoral neck and lumbar spine were higher in MetS participants compared to non-MetS participants 11. Similarly, Maghraoui et al. reported that women with MetS had significantly greater BMD at the hip and spine and a lower prevalence of osteoporosis (17.7% vs. 34.1%) than those without MetS 22. In a recent study of 1587 Arab adults, the authors found a significant positive correlation between MetS and BMD at the spine, regardless of gender 23. There are also studies in which the opposite results of this positive correlation were obtained. In a study including 2475 Korean women, Hwang et al. observed an association between low spine BMD and MetS 24. In some studies, a possible gender difference in the relationship between MetS and bone has also been observed, as MetS is a risk factor for low BMD in men, but may not ba significant predictor for women 7 25. The authors explained these results with some gender differences in fat deposition and hypothesized that the mechanical effect and estrogen synthesis were more prominent in women, and the bone-damaging fat-related factors associated with oxidative stress and chronic inflammation were more prominent in men.

MetS is a multidimensional syndrome consisting of several individual components, each of which may affect BMD and bone turnover 6. Among the MetS components, a significant positive correlation (weak-to-moderate) was observed between abdominal obesity and BMD at the femoral neck and spine in this study (r=0.375, 0.340 respectively, p<0.001). In addition, a weak but significant positive correlation was observed between other components (triglycerides, systolic and diastolic blood pressure) of MetS and BMD (Table 3). It is hypothesized that hyperinsulinemia and peripheral aromatization of sex hormones cause abdominal fat accumulation in MetS patients 25. In our study population, all women were overweight with a median BMI of 30.9. However, the median BMI was higher in women without osteoporosis than in women with osteoporosis (32 vs 28.7, p<0.001). The positive association between abdominal obesity and BMD was first reported by Edelstein et al. 26 in 1999. Excessive body weight is thought to have this effect on BMD through mechanical loading 25. Some studies have shown a significant association between central obesity and low BMD 27, 28, and some have also reported a potential gender difference . The researchers explain that the differences between men and women may be due to gender-related differences in fat deposition, as mentioned above. More studies are needed to explore the relationship between central obesity and BMD.

Osteoblasts and adipocytes share common progenitor cells in the bone marrow, so it is thought that body fat-related components such as in MetS may be associated with BMD 29. In our study population, a positive correlation was shown between serum triglycerides and spine BMD. Cui et al. 30 reported a positive correlation between serum triglycerides and BMD at the trochanter region in Korean postmenopausal women. However, similar to our results, the researchers did not observe any correlation between HDL-C levels and BMD values in any of the regions. There are also studies supporting that high triglyceride levels are associated with low BMD 24, 31. High levels of triglycerides are stored in the body as adipose tissue, so this positive association may be confused with increased body weight. It is also hypothesized that triglycerides regulate bone metabolism with apolipoprotein A 32. In some clinical studies, lipids have been shown to play a potential role in osteoporosis. There are also studies indicating that statins are associated with increased BMD and reduced fracture risk 33, 34. Dawood et al. observed that patients with osteoporosis had low HDL-C levels 35. In this study, serum HDL-C levels were similar in women with and without osteoporosis.

One of the most important effects of MetS on BMD is the reduction of blood circulation to bone mass because of micro-vascular complications related to poor glucose regulation 6. In current studies, there is strong evidence supporting that Type 1 DM causes a decrease in BMD, while the effects of Type 2 DM on bone density remain unclear 16. In our study, correlation analysis revealed a weak but significant positive association between FBG and spine BMD. However, FBG was not identified as an independent factor influencing BMD in multiple regression analysis. The limited number of patients, as mentioned earlier, may have led to such a result. In a study by Tseng et al. 36, it was shown that there was no significant relationship between FBG and BMD. In some studies, variation in FBG is related to high BMD 7 16, 27. Insulin is shown anabolic effects on the bone with insulin-like growth factor-1, and increased insulin secretion to impaired glucose regulation in MetS may trigger bone formation 37.

Correlation analysis revealed a positive correlation between systolic blood pressure and BMD at the spine and femoral neck in this study. However, in linear regression analysis, this association was significant only for spine BMD. Diastolic blood pressure was also associated with spine BMD. In some studies, hypertension is related to low BMD as a consequence of increased serum parathyroid hormone (PTH) levels or urinary calcium excretion, but the results are conflicting 16. Hanley et al. 38 observed a significant relationship between hypertension and higher BMD for both men and women. Mussolino et al. 39 reported no relationship between blood pressure and BMD. Yang et al. 40 demonstrated that women with hypertension have a lower BMD at the femoral neck and that hypertension is also an independent risk factor for fragility fracture in women. Similar to this study, Yarema et al. 41 reported that the T-score values of BMD in post-menopausal women with hypertension were significantly lower than in women without hypertension.

We hope that this study will help determine whether MetS is an important risk factor for osteoporosis in post-menopausal Turkish women. In addition, this study will be useful in demonstrating the potential effects of MetS components on BMD. However, the study has several limitations. First, this was a hospital-based study, so the sample size was limited. More population-based studies are needed in this field. Second, selection bias cannot be ruled out in this study. The study population consisted of volunteer participants for health screening and participants referred by a physician for BMD testing. Therefore, these individuals may be more conscious about their health, and they may not be representative of the general population. Lastly, it was a cross-sectional study, and the cause-effect relationship of BMD with MetS needs to be explored with prospective studies.

In conclusion, we observed in this study that MetS is associated with increased BMD at the femoral neck and spine in postmenopausal women, possibly due to increased mechanical loading on the bone. Regarding the relationship between MetS components and BMD, a positive significant correlation was observed between waist circumference and BMD in both spine and femoral neck, and also a weak but significant positive association was observed between serum triglyceride levels, systolic and diastolic blood pressure, and spine BMD. In line with these results, it can be said that MetS is not an important risk factor for osteoporosis. Considering inconsistent results in this field, it is clear that more multicenter population-based studies are needed to clarify the relationship between MetS and its components on bone health.
